# Supplementary material for: International lineages of Salmonella enterica serovars isolated from chicken farms, Wakiso District, Uganda
Source: PLoS One. 2020 Jan 28;15(1):e0220484. doi: 10.1371/journal.pone.0220484 (PMC6986767; doi:10.1371/journal.pone.0220484)
Supplement: Attachment — Submitted filename: Comments on International lineages of Salmonella manuscript.pdf [file pone.0220484.s001.pdf]

ABSTRACT

The growing occurrence of multidrug-resistant (MDR) *Salmonella enterica* in poultry has been reported with public health concern worldwide. We reported, recently, the occurrence of *Escherichia coli* and *Salmonella enterica* serovars carrying clinically relevant resistance genes in dairy cattle farms in the Wakiso District, Uganda, highlighting an urgent need to monitor food-producing animal environments. Here, we present the prevalence, antimicrobial resistance, and sequence type of 51 *Salmonella* isolates recovered from ~~400~~ environmental samples from chicken farms in Uganda. Among the *Salmonella* isolates, 32/51 (62.7%) were resistant to at least one antimicrobial, and 10/51 (19.6%) displayed multiple drug resistance. Through PCR, five replicon plasmids were identified among all chicken *Salmonella* including *Inc*FIIS 17/51 (33.3%), *Inc*I1α 12/51 (23.5%), *Inc*P 8/51 (15.7%), *Inc*X1 8/51 (15.7%), and *Inc*X2 1/51 (2.0%). In addition, we identified replicons through WGS (ColpVC and *Inc*FIB). A significant seasonal difference between chicken sampling periods was observed (p= 0.0017). We conclude that MDR *Salmonella* highlights the risks posed to the animals, environment, and humans for infection. Implementing a robust integrated surveillance system ~~in Uganda~~ will help monitor MDR to help control infectious threats.

Introduction

Multidrug-resistant (MDR) *Salmonella enterica* remains a major public health concern being reported in food, animal, human and environmental settings, particularly in developing countries. Additionally, international lineages have been readily spread

worldwide (1-6), leading a high impact on public health, which has been deemed a global pressure (WHO).

In Uganda, antibiotics are increasingly being used and not monitored or regulated in food-producing animals. This practice is well established to select antibiotic-resistant strains that can spread to humans through the food chain. In this concern, considering the lack of information regarding antimicrobial resistance (AMR) in developing countries, Uganda has plans for an integrated national surveillance system for foodborne pathogen which is included in the National Action Plan (NAP) on AMR, using a One Health approach (7).

Therefore, we present a cross-sectional study developed in chicken farms, in Uganda to investigate the prevalence, AMR, and their genomic aspects of *Salmonella enterica* serovars.

Methods

Bacterial Isolates: In our previous study, we reported on the phenotypic characterization of *Salmonella* isolates from cattle farms. We also collected *Salmonella* isolates from chicken farms in parallel to the cattle farms (5). This study was designed as two cross-sectional studies over one year. Sampling occurred over two seasons, the rainy season that began in March ending in September, and the dry season that began in June ending in December. Enrollment in the study occurred through contact with producers throughout the Wakiso district. A total of 20 chicken producers (20 farms) agreed to participate in the study. On-farm sampling was conducted once during the rainy and dry seasons totaling 39 collection periods (two farms dropped out of study in the rainy season). Ten

samples per farm were collected at each visit totaling 379 samples (one farm had nine samples).

Ten drag swabs were used per farm. Drag swabs (3" x 3" sterile gauze pads) in sterile skim milk was the preferred collection tool (Hardy Diagnostics, Inc., Santa Maria, CA). A sampling schematic was pre-drawn to ensure maximum sampling of the house floor environment, including inside diagonals, feeding and water containers, coops, and wall to wall samples. Swabs were individually placed in a sterile whirl-pak bag; the bag was kept on ice in a cooler prior to transport to the laboratory. Isolation of *Salmonella* was collected as previously described in Fedorka-Cray et al. (8).

Antimicrobial Resistance testing: A total of 51 *Salmonella* were isolated from chicken farms and tested for AMR using the National Antimicrobial Resistance Monitoring System (NARMS) gram-negative panels (Thermo Fisher Scientific Inc, Waltham, MA) as described by Ball et al. (5). All 51 isolates were frozen in LB broth with 30% glycerol (Thermo Fisher Scientific Inc, Waltham, MA) at -80°C.

Molecular characterization: The 51 *Salmonella* isolates were struck for isolation from the frozen stocks to Tryptic Soy Agar (TSA) with 5% sheep blood (BAP) (Thermo Fisher Scientific Inc, Waltham, MA) and incubated overnight at 37°C to ensure purity. Lysates were prepared by suspending a loopful of well-isolated colonies into 200 µl of molecular grade water and vortexed at maximum speed for several seconds. The suspension was boiled at 100°C for 10 minutes, centrifuged at 13 X 1000 rpm for 60 seconds, and the supernatant was collected for use as the DNA template. For PCR screening and whole genome sequencing, all methods were followed as described in Ball et al. (manuscript submitted).

Whole-genome sequencing: DNA extraction was performed using a commercial kit (QiAmp tissue, Qiagen, Germany) according to manufacturer's guidelines. Genomic DNA (*n*= 51) were sequenced at a 300-bp paired-end-read using the Nextera XT library preparation kit at the MiSeq platform (Illumina, San Diego, CA). *De novo* assembly was achieved using CLC Genomics Workbench 10.1.1 (Qiagen). Resistome, plasmidome and multilocus sequence typing (MLST) were identified using multiple databases as ResFinder 3.1, PlasmidFinder 2.0, and MLST 2.0, respectively, available from the Center for Genomic Epidemiology (http://genomicepidemiology.org/). Sequence data were deposited in the GenomeTrakr Project.

Statistical Analysis: The prevalence of *Salmonella* were analyzed using WHONET and Microsoft Excel. A logistic regression model was used in SAS® software (SAS® Cary, NC) where season (rainy and dry) served as the factor. Farm was included as a random effect.

Results

Table 1 displays the results of serotype, AMR phenotype, AMR genotype, and plasmid identification. Fifty-one *Salmonella* were isolated (51/379; 13.5%) from chicken belonging eight different serotypes in order of highest to lowest, *Salmonella* serovar Enteritidis (31.3%); *S.* Kentucky (21.6%); *S.* Zanzibar and *S.* Virchow (15.7%); *S.* Newport and *S.* serovar 42:r:- (5.88%), *S.* Typhimurium (4%) and *S.* Barranquilla at 2.0%. The prevalence of *Salmonella* was statistically significantly higher in the rainy season (p=0.0017).

The AMR phenotype displayed resistance to eight antimicrobials including tetracylcine (51%), nalidixic acid (37.3%), sulfisoxazole (23.5%), ciprofloxacin (21.6%),

streptomycin (13.7%), ampicillin (7.8%), sulfamethoxazole (3.9%), chloramphenicol (2%). Whole genome sequencing analysis revealed the presence of resistance genes to tetracycline [*tetA*; 53%], sulfonamides [*sul2* (21.5%); *sul3* (11.7%)], streptomycin [*strA* (19.6%); *strB* (19.6%)], aminoglycosides [*aph(6)-Id* (15.6%); *aph(3")-Ib* (11.7%); *aadA1* (11.7%); *aadA2* (2%)], β-lactams [bla_{TEM-1B}; 9.8%], quaternary ammonium [*qacL*; 5.8%], quinolones [*qnrS1*; 5.8%] and trimethoprim [*dfrA14*; 4%]. Other than acquiring resistance genes were assigned as quinolone resistance determining regions (QRDR) with point mutation in *gyrA* and *parC* as we can observe in Table 1. Ten isolates (19.6%) showed DNA gyrase (GyrA-S83F-D87N) with a double amino acid mutation in GyrA, serine to phenylalanine at codon 83 and aspartic acid to asparagine at 87, whereas eight isolates (15.6%) showed a single amino acid substitution of serine to tyrosine at codon 83. For QRDR in *parC* was observed (n=10; 19.6%) only one substitution in serine to isoleucine at codon 80. No mutations were found in *gyrB* and *parE*.

Afterward, the prevalence of plasmids related to resistance or virulence factors were screened through sequences. Six plasmids were identified being IncFII(S)-IncFIB (S)-ColpVC the most commons distributed in S. Enteritidis; Incl1-ColpVC in *S.* Kentucky and *S.* Zanzibar; IncX2 in *S.* Newport; Incl1-IncFII(S)-IncFIB (S)-ColpVC in *S.* Typhimurium and Col440I in *S.* serovar 42:r:-.

In addition, nine sequence types (ST) such as ST11, ST198, ST466, ST16, ST166, ST46, ST19, ST1208 and ST3807 were associated with *S.* Enteritidis, *S.* Kentucky, *S.* Zanzibar, *S.* Virchow, *S.* Newport, *S.* Newport, *S.* Typhimurium, *S.* serovar 42:r:- and *S.* Barranquilla, respectively.

Table 1: Antimicrobial resistance phenotype and genotype comparison of *Salmonella* from chickens in the Wakiso district of Uganda (n=51)

Sample ID	Serovar	ST	Resistance profile (MIC)	Resistance genes	*gyrA*	*parC*	Plasmids	Plasmids (PCR)
SALM-44	42:r:-	1208	STR	Pansusceptible	none	none	Col440I	none
SALM-46	42:r:-	1208	STR	Pansusceptible	none	none	none	none
SALM-51	42:r:-	1208	STR	Pansusceptible	none	none	none	none
SALM-47	Barranquila	3807	Pansusceptible	Pansusceptible	none	none	none	none
SALM-1	Enteritidis	11	Pansusceptible	*strA, strB, aadA1, blaTEM-1B, sul2, sul3, tetA*	none	none	IncFII(S), IncFIB (S), ColpVC	IncFII(S)
SALM-2	Enteritidis	11	Pansusceptible	Pansusceptible	none	none	IncFII(S), IncFIB (S), ColpVC	IncFII(S)
SALM-3	Enteritidis	11	Pansusceptible	*sul2*	none	none	IncFII(S), IncFIB (S), ColpVC	IncFII(S)
SALM-5	Enteritidis	11	Pansusceptible	Pansusceptible	none	none	IncFII(S), IncFIB (S), ColpVC	IncFII(S)
SALM-7	Enteritidis	11	Pansusceptible	Pansusceptible	none	none	IncFII(S), IncFIB (S), ColpVC	IncFII(S)
SALM-26	Enteritidis	11	Pansusceptible	Pansusceptible	none	none	IncFII(S), IncFIB (S)	IncFII(S)
SALM-28	Enteritidis	11	Pansusceptible	Pansusceptible	none	none	IncFII(S), IncFIB (S)	IncFII(S)
SALM-29	Enteritidis	11	Pansusceptible	Pansusceptible	none	none	IncFII(S), IncFIB (S)	IncFII(S)
SALM-30	Enteritidis	11	Pansusceptible	Pansusceptible	none	none	IncFII(S), IncFIB (S)	IncFII(S)
SALM-31	Enteritidis	11	Pansusceptible	Pansusceptible	none	none	IncFII(S), IncFIB (S)	IncFII(S)
SALM-32	Enteritidis	11	Pansusceptible	Pansusceptible	none	none	IncFII(S), IncFIB (S)	IncFII(S)
SALM-33	Enteritidis	11	Pansusceptible	Pansusceptible	none	none	IncFII(S), IncFIB (S)	IncFII(S)
SALM-41	Enteritidis	11	Pansusceptible	Pansusceptible	none	none	IncFII(S), IncFIB (S), Col440I	none
SALM-42	Enteritidis	11	Pansusceptible	Pansusceptible	none	none	none	IncFII(S)
SALM-43	Enteritidis	11	Pansusceptible	Pansusceptible	none	none	IncFII(S), IncFIB (S), Col440I	IncFII(S)
SALM-49	Enteritidis	11	Pansusceptible	Pansusceptible	none	none	IncFII(S), IncFIB (S)	none

20

indicate in the table which isolates were from the rainy season. Two tables?

Table 1 cont'd

Sample ID	Serovar	ST	Resistance profile (MIC)	Resistance genes	*gyrA*	*parC*	Plasmids	Plasmids (PCR)
SALM-8	Kentucky	198	AMP, CIP, NAL, STR, SOX, TCY, SXT	*aadA1, aph(6)-Id, strA, strB, blaTEM-1B, dfrA14, qacL, sul2, sul3, tet(A)*	S83F/D87N	S80I	ColpVC, IncI1	IncI1α
SALM-9	Kentucky	198	CIP, NAL	Pansusceptible	S83F/D87N	S80I	ColpVC	none
SALM-10	Kentucky	198	CIP, NAL, STR, SOX, TCY	*aph(3")-Ib, aph(6)-Id, strA, strB, sul2, tet(A)*	S83F/D87N	S80I	ColpVC	none
SALM-11	Kentucky	198	CIP, NAL, STR, SOX, TCY	*aph(3")-Ib, aph(6)-Id, strA, strB, sul2, tet(A)*	S83F/D87N	S80I	ColpVC	none
SALM-12	Kentucky	198	CIP, NAL, STR, SOX, TCY	*aph(3")-Ib, aph(6)-Id, strA, strB, sul2, tet(A)*	S83F/D87N	S80I	ColpVC	none
SALM-13	Kentucky	198	CIP, NAL, STR, SOX, TCY	*aph(3")-Ib, aph(6)-Id, strA, strB, sul2, tet(A)*	S83F/D87N	S80I	ColpVC	none
SALM-14	Kentucky	198	CIP, NAL, STR, SOX, TCY	*aph(3")-Ib, aph(6)-Id, strA, strB, sul2, tet(A)*	S83F/D87N	S80I	ColpVC	none
SALM-15	Kentucky	198	CIP, NAL, STR, SOX, TCY	*aph(3")-Ib, aph(6)-Id, strA, strB, sul2, tet(A)*	S83F/D87N	S80I	ColpVC	none
SALM-16	Kentucky	198	CHL, CIP, NAL, STR, SOX, TCY, SXT	*qnrS1, aadA1, aadA2, aph(6)-Id, strA, strB, cmlA1, dfrA14, sul2, sul3, tet(A)*	S83F/D87N	S80I	ColpVC, IncI1	IncI1α
SALM-17	Kentucky	198	CIP, NAL, STR, SOX, TCY	*strA, strB, sul2, tetA*	none	none	ColpVC	none
SALM-18	Kentucky	198	AMP, CIP, NAL, SOX	*aadA1, blaTEM-1B, sul3*	S83F/D87N	S80I	ColpVC, IncI1	IncI1α
SALM-45	Newport	166	NAL, TCY	*qnrS1, tetA*	none	none	IncX2	IncX2
SALM-50	Newport	46	Pansusceptible	Pansusceptible	none	none	none	none
SALM-4	Typhimurium	19	AMP, SOX	*aadA1, blaTEM-1B, qacL, sul3*	none	none	IncI1, IncFII(S), IncFIB (S), ColpVC	IncI1α, IncFII(S)
SALM-6	Typhimurium	19	AMP, SOX	*aadA1, blaTEM-1B, qacL, sul3*	none	none	IncI1, IncFII(S), IncFIB (S), ColpVC	IncI1α, IncFII(S)
SALM-34	Virchow	16	NAL, TCY	*tetA*	S83Y	none	none	IncP, IncX1
SALM-35	Virchow	16	NAL, TCY	*tetA*	S83Y	none	none	IncP, IncX1

37

Table 1 cont'd

Sample ID	Serovar	ST	Resistance profile (MIC)	Resistance genes	*gyrA*	*parC*	Plasmids	Plasmids (PCR)
SALM-36	Virchow	16	NAL, TCY	*tetA*	S83Y	none	none	IncP, IncX1
SALM-37	Virchow	16	NAL, TCY	*tetA*	S83Y	none	none	IncP, IncX1
SALM-38	Virchow	16	NAL, TCY	*tetA*	S83Y	none	none	IncP, IncX1
SALM-39	Virchow	16	NAL, TCY	*tetA*	S83Y	none	none	IncP, IncX1
SALM-40	Virchow	16	NAL, TCY	*tetA*	S83Y	none	none	IncP, IncX1
SALM-48	Virchow	16	NAL, TCY	*tetA*	S83Y	none	none	IncP, IncX1
SALM-19	Zanzibar	466	TCY	*tetA*	none	none	IncI1	IncI1α
SALM-20	Zanzibar	466	TCY	*tetA*	none	none	IncI1	IncI1α
SALM-21	Zanzibar	466	TCY	*tetA*	none	none	ColpVC, IncI1	IncI1α
SALM-22	Zanzibar	466	TCY	*tetA*	none	none	IncI1	IncI1α
SALM-23	Zanzibar	466	TCY	*tetA*	none	none	ColpVC, IncI1	IncI1α
SALM-24	Zanzibar	466	Pansusceptible	Pansusceptible	none	none	ColpVC	IncI1α
SALM-25	Zanzibar	466	TCY	*tetA*	none	none	IncI1	IncI1α
SALM-27	Zanzibar	466	TCY	*tetA*	none	none	IncI1	IncI1α

Discussion

The percent prevalence of *Salmonella* (13.5%) in this study highlights the potential risk to the cross-contamination between human and poultry in Ugandan households. There are limited reports on the prevalence of *Salmonella* on chicken farms and the reports that are available show very little resistance compared to this study. Afema et al. reported 6.6% *Salmonella* was detected in live birds markets within Kampala, Uganda (9). We also learned that there was a seasonal effect in the recovery of *Salmonella*. Uganda typically has a rainy season that occurs between March to May and October to December (10). For recovery from chicken farms, a significant difference (p=0.0017) for recovery of *Salmonella* between the rainy and dry seasons as a higher prevalence of *Salmonella* was observed. During the rainy season, there is an increase in humidity as well as moisture which has been reported to influence the recovery of several bacterial species in poultry (11).

The serotype distribution in this study indicated that *Salmonella* serovars Enteritidis and Kentucky were most often recovered from chicken samples. This is comparable to the most commonly seen serotypes in chickens reported in the US (12). Kentucky has previously been reported in Uganda in humans, poultry, and the environment (9).

Among chicken isolates, *Salmonella* presented with MDR phenotypes to the antimicrobials tested. Approximately 38% of the isolates were resistant to two or more classes of antimicrobials, including two isolates resistant to seven antimicrobials. The *Salmonella* serovar Kentucky isolates in this study presented MDR to over five (ciprofloxacin, nalidixic acid, streptomycin, sulfisoxazole, and tetracycline) or seven

(chloramphenicol, ampicillin, ciprofloxacin, nalidixic acid, streptomycin, sulfisoxazole, tetracycline, and trimethoprim-sulfamethoxazole) antimicrobials. All *Salmonella* serovar Kentucky isolates resistant to ciprofloxacin. Since the early 2000s, ciprofloxacin resistance for *Salmonella* serovar Kentucky has been on the rise, especially from travelers to northern and eastern Africa (13). Rickert-Hartman et al. found that 9% of the *Salmonella* serovar Kentucky isolated from travelers were ciprofloxacin resistant. An interesting note was that poultry was thought to be a reservoir for these resistant strains (13, 14). Cases of ciprofloxacin-resistant Kentucky have been seen in the US from travelers from India, resulting in seven infected with one death (13). In this regard, the emergence of *S.* Kentucky ST198 pose a major threat to public health worldwide, particularly for being highly drug-resistant (15) and has been reported in different sources including retail chicken carcasses (16). Additionally, the presence of chromosome mutation can be useful for tracking the pandemic ciprofloxacin-resistant S. Kentucky strain ST198 from geographically distinct regions (15).

We further characterized these isolates with WGS to see if concordance was seen and if isolates presented β-lactamase resistance genes. TEM-1B was identified in five isolates that PCR methods did not identify. In previous studies (17), discrepancies were seen between phenotypic resistance and genotypic analysis using WGS. It was reported that a MIC might not reach the breakpoint, but resistance genes were present (17).

Five of the 28 plasmids that were screened through PCR were observed in multiple isolates: *Inc*FIIS (17/51; 33.3%), *Inc*I1α (12/51; 23.5%), *Inc*P (8/51; 15.7%), *Inc*X1 (8/51; 15.7%), and *Inc*X2 (1/51; 2.0%). After analyzing the WGS sequences for plasmids, we notice a difference in the plasmids that were identified. In 12 isolates, there

was concordance with the IncI1α with seven of the 12 having an additional plasmid (ColpVC) that was not screened in the PCR and two with *Inc*FIIS plasmid. Seventeen isolates were in concordance with the *Inc*FIIS plasmid. These same 17 isolates also presented *Inc*FIB (S) plasmids, and ColpVC and Col4401 were identified in seven and two isolates, respectively. *Inc*X2 and *Inc*P were not identified in the WGS analysis as was in the PCR. Ten isolates were negative for PCR, but WGS identified as ColpVC (nine isolates) and Col4401 (one isolate).

*Inc*FIIS was the most common plasmid identified in this study at 33.3% (17/51). Studies have shown that bacterial isolates containing *bla*$_{CTX\text{-}M\text{-}1}$, harbor the *Inc*FIIS along with other incompatibility plasmids (18). *Inc*1 plasmids are known to be distributed throughout many serotypes of *Salmonella* and predominate in both *E.coli* and *Salmonella* (19-21). In this study, *Inc*1α was observed among *Salmonella* serovars such as Zanzibar, Kentucky, and Typhimurium. All isolates from *Salmonella* serovar Kentucky came from the same farm, as well as isolates with *Salmonella* serovar Typhimurium.

*Inc*P and *Inc*X1 were the next most common plasmids seen in this study through PCR. Both were present in the *Salmonella* serovar Virchow isolates. It has been reported that *Inc*P can spread through groups of bacteria via conjugative transfer and code for broad range antimicrobial resistance. *Inc*P is highly likely to be found in manure, wastewater, and soil (22). *Inc*X1 is commonly found as a narrow host-range plasmid in *Enterobacteriaceae,* also spreading to other bacteria via conjugative transfer (23).

Conclusion

In summary, we present in this study the clonal distribution of eight *Salmonella* enterica serovars displaying resistance to clinically important antibiotics. Of these, the

presence of international lineages as ciprofloxacin-resistant *S.* Kentucky sequence type 198 in chicken farms raises a public concern; given that fluoroquinolones are the first treatment choice. Our findings suggest that endemic dissemination of resistant serovars, adding valuable information in the epidemiological surveillance in Uganda. Therefore, these results may encourage addition genomic surveillance studies in this region to aid the development of mitigation strategies to limit the global distribution of these multi-drug resistant *Salmonella enterica*.

Acknowledgments

We would like to acknowledge our funding sources from North Carolina State University (NCSU), College of Veterinary Medicine and the WHO AGISAR Secretariat; Our colleagues at Makerere University Dr. Eddie Wampande, Sarah Tegule, Samuel Maling, David Apollo Munanura, Allan Odeke, Disan Muhangazi, Mark Ogal, Mutumba Paul, and Elizabeth Basemera; Our colleagues at the NCSU, College of Veterinary Medicine Diagnostic Laboratory, Dr. Megan Jacob.

References

1. Djeffal S, Mamache B, Elgroud R, Hireche S, Bouaziz O. Prevalence, and risk factors for *Salmonella* spp. contamination in broiler chicken farms and slaughterhouses in the northeast of Algeria. Veterinary world. 2018;11(8):1102-8.
2. Elnekave E, Hong S, Mather AE, Boxrud D, Taylor AJ, Lappi V, et al. *Salmonella* enterica Serotype 4,[5],12:i:- in Swine in the United States Midwest: An Emerging Multidrug-Resistant Clade. Clinical infectious diseases: an official publication of the Infectious Diseases Society of America. 2018;66(6):877-85.
3. Li XP, Fang LX, Song JQ, Xia J, Huo W, Fang JT, et al. Clonal spread of mcr-1 in PMQR-carrying ST34 *Salmonella* isolates from animals in China. Scientific reports. 2016;6:38511.
4. Nadimpalli M, Fabre L, Yith V, Sem N, Gouali M, Delarocque-Astagneau E, et al. CTX-M-55-type ESBL-producing *Salmonella* enterica are emerging among retail meats in Phnom Penh, Cambodia. The Journal of antimicrobial chemotherapy. 2019;74(2):342-8.

5. Ball TA, Monte DF, Aidara-Kane A, Matheu-Alvarez J, Ru H, Thakur S, et al. Phenotypic and Genotypic Characterization of *Escherichia coli* and *Salmonella* enterica from Dairy Cattle Farms in the Wakiso District, Uganda: A Cross-Sectional Study. Foodborne pathogens and disease. 2019;16(1):54-9.
6. Toro M, Retamal P, Ayers S, Barreto M, Allard M, Brown EW, et al. Whole-Genome Sequencing Analysis of *Salmonella* enterica Serovar Enteritidis Isolates in Chile Provides Insights into Possible Transmission between Gulls, Poultry, and Humans. Applied and environmental microbiology. 2016;82(20):6223-32.
7. UNAS, CDDEP, GARP-Uganda, Mpairwe Y, Wamala S. Antibiotic Resistance in Uganda: Situation Analysis and Recommendations. Kampala, Uganda: Uganda National Academy of Sciences; Center for Disease Dynamics, Economics & Policy; 2015.
8. Fedorka-Cray PJ, Bush E, Thomas L, Gray J, McKean J. *Salmonella* Infection in Herds of Swine. 1996.
9. Afema JA, Byarugaba DK, Shah DH, Atukwase E, Nambi M, Sischo WM. Potential Sources and Transmission of *Salmonella* and Antimicrobial Resistance in Kampala, Uganda. PloS one. 2016;11(3):e0152130.
10. Kigozi R, Zinszer K, Mpimbaza A, Sserwanga A, Kigozi SP, Kamya M. Assessing temporal associations between environmental factors and malaria morbidity at varying transmission settings in Uganda. Malar J. 2016;15(1):511.
11. Akil L, Ahmad HA, Reddy RS. Effects of climate change on *Salmonella* infections. Foodborne pathogens and disease. 2014;11(12):974-80.
12. United States Department of Agriculture. Serotypes Profile of *Salmonella* Isolates from Meat and Poultry Products, January 1998 through December 2014 2014 [Available from https://www.fsis.usda.gov/wps/portal/fsis/topics/data-collection-and-reports/microbiology/annual-serotyping-reports.
13. Rickert-Hartman R, Folster JP. Ciprofloxacin-resistant *Salmonella* enterica serotype Kentucky sequence type 198. Emerg Infect Dis. 2014;20(5):910-1.
14. Weill FX, Bertrand S, Guesnier F, Baucheron S, Cloeckaert A, Grimont PA. Ciprofloxacin-resistant *Salmonella* Kentucky in travelers. Emerg Infect Dis. 2006;12(10):1611-2.
15. Le Hello S, Harrois D, Bouchrif B, Sontag L, Elhani D, Guibert V, et al. Highly drug-resistant *Salmonella* enterica serotype Kentucky ST198-X1: a microbiological study. The Lancet Infectious Diseases. 2013;13(8):672-9.
16. Ramadan H, Gupta SK, Sharma P, Sallam KI, Hiott LM, Elsayed H, et al. Draft genome sequences of two ciprofloxacin-resistant *Salmonella* enterica subsp. enterica serotype Kentucky ST198 isolated from retail chicken carcasses in Egypt. Journal of global antimicrobial resistance. 2018;14:101-3.
17. McDermott PF, Tyson GH, Kabera C, Chen Y, Li C, Folster JP, et al. Whole-Genome Sequencing for Detecting Antimicrobial Resistance in Nontyphoidal *Salmonella*. Antimicrobial agents and chemotherapy. 2016;60(9):5515-20.
18. Zurfluh K, Jakobi G, Stephan R, Hachler H, Nuesch-Inderbinen M. Replicon typing of plasmids carrying bla CTX-M-1 in Enterobacteriaceae of animal, environmental and human origin. Front Microbiol. 2014;5:555.
19. Dierikx C, van Essen-Zandbergen A, Veldman K, Smith H, Mevius D. Increased
