# Supplementary material for: International lineages of Salmonella enterica serovars isolated from chicken farms, Wakiso District, Uganda
Source: PLoS One. 2020 Jan 28;15(1):e0220484. doi: 10.1371/journal.pone.0220484 (PMC6986767; doi:10.1371/journal.pone.0220484)
Supplement: Attachment — Submitted filename: The article by Ball et al.pdf [file pone.0220484.s003.pdf]

The article by Ball et al. tackles the vital subject of antimicrobial resistance food-producing animals in a region from where very little is known regarding this subject. It is a welcome addition to the body of knowledge, although the article is missing information and data analysis.

1- The farms a poorly described both, in a geographical sense and relation to each other. Furthermore, the type of production, size, and information on different types of animals on the farm are not mentioned but are necessary.
2- Spatial and temporal information on the tables is missing, and it is barely described in the results, and not discussed.
3- The presence of resistance genes without phenotypic testing is an indication of resistance, but the isolates cannot be described as resistant as it is the case in the discussion.
5- Information on the quality of the sequencing obtained for each isolate should be documented. If they met Genometrackr standards, please estate this fact, and their current minimum.
4-Exact information on the bioinformatics pipeline used to analyze the samples Is crucial for each isolate. Where different databases used every time? Where there any differences between the databases?
