# Supplementary material for: International lineages of Salmonella enterica serovars isolated from chicken farms, Wakiso District, Uganda
Source: PLoS One. 2020 Jan 28;15(1):e0220484. doi: 10.1371/journal.pone.0220484 (PMC6986767; doi:10.1371/journal.pone.0220484)
Supplement: Attachment — Submitted filename: Review Salmonella manuscript.docx [file pone.0220484.s002.docx]

The manuscript would be more insightful if the authors would have been able to find out which antibiotics were used on the farms the samples came from. Are there any data on which serovars are common in humans in Uganda? Are the MDR levels higher or lower than those observed in other parts of the world?

The manuscript contains numerous language issues and unclear sentences. Below are suggestions for improvements and questions that should be addressed.

L.25: The growing occurrence of multidrug-resistant (MDR) Salmonella enterica in poultry is a public health concern worldwide. The present study, the prevalence, antimicrobial resistance, and sequence type [sequence of what?] of 51 Salmonella isolates recovered from 400 environmental samples from chicken farms in Uganda.

L. 33: Five plasmid replicons were identified among all isolates, including IncFIIS 17/51 35 (33.3%), IncI1α 12/51 (23.5%), IncP 8/51 (15.7%), IncX1 8/51 (15.7%), and IncX2 1/51 (2.0%). In addition, ColpVC and IncFIB replicons were identified through WGS [spell out].

L. 45: Additionally, international lineages [what are these?] have been readily spread …

L. 48: In Uganda, antibiotics [what kind?] are increasingly being used [based on what information?] and not monitored or regulated in food-producing animals.

L. 50: To address this concern and considering the lack of information regarding antimicrobial resistance (AMR) in developing countries, Uganda ….

L. 55: Therefore, we present a cross-sectional study involving chicken farms in Uganda to investigate the prevalence, AMR, and their?? [referring to what?] genomic aspects [what is meant by “aspects”?] of Salmonella enterica serovars.

L. 60: Salmonella isolates were collected from chicken farms in parallel with the collection from cattle farms (5) as part of a cross-sectional study spanning one year.

L. 76: Isolation of Salmonella was done as described by Fedorka-Cray et al. (8).

L. 81: [Move this sentence to the previous section.] The isolates were frozen in LB broth with 30% glycerol (Thermo Fisher Scientific Inc, Waltham, MA) at -80o C.

L. 78: [Combine the sections.] Antimicrobial Resistance testing and molecular characterization of …[what?]

A total of 51 Salmonella were obtained from chicken farms. For analyses, the isolates were retrieved from frozen stock, plated on Tryptic Soy Agar (TSA) with 5% sheep blood (BAP) (Thermo Fisher Scientific Inc, Waltham, MA) and incubated overnight at 37oC. AMR testing was done using the National Antimicrobial Resistance Monitoring System (NARMS) gram-negative panels (Thermo Fisher Scientific Inc, Waltham, MA) as described by Ball et al. (5). Lysates were prepared by suspending a loopful of well-isolated colonies in 200 µl of molecular grade water and vortexing at maximum speed for several seconds. The suspension was boiled at 100°C for 10 minutes, centrifuged at ..x g for 60 seconds, and the supernatant was collected for use as the DNA template. PCR screening [for what?] and whole genome sequencing were carried out as described in Ball et al. (manuscript submitted). [Details should be provided in case publication is delayed.]

L. 96: Resistomes, plasmidomes and multilocus sequence types (MLST) were identified using ResFinder 3.1, PlasmidFinder 2.0, and MLST 2.0, respectively, available from the Center for Genomic Epidemiology (http://genomicepidemiology.org/). Sequence data were deposited in the GenomeTrakr Project. [I could not find any data on a project from Uganda. Please make sure it is available as you state.]

L. 107: Fifty-one Salmonella were isolated (51/379; 13.5%) belonging eight different serovars: Enteritidis (31.3%); S. Kentucky (21.6%); S. Zanzibar and S. Virchow (15.7%); S. Newport and 42:r:- (5.88%), Typhimurium (4%) and S. Barranquilla (2.0%). The prevalence of Salmonella was higher in the rainy season (p=0.0017). The isolates displayed resistance to eight antimicrobials including tetracylcine (51%), ….

L. 120: Other than acquiring resistance genes???[I do not understand this part of the sentence.] were assigned as quinolone resistance determining regions (QRDR) with point mutation in gyrA and parC (Table 1). Ten isolates (19.6%) showed a double amino acid mutation in GyrA (GyrA-S83F-D87N), whereas eight isolates (15.6%) showed a single amino acid substitution of serine to tyrosine at codon 126. For QRDR in parC (n=10; 19.6%) only one substitution of serine by isoleucine at codon 80 was observed. No mutations were found in gyrB and parE. Sequencing identified six plasmids. IncFII(S)-IncFIB (S)- ColpVC were the most common in S. Enteritidis; Incl1-ColpVC in S. Kentucky and S. Zanzibar; IncX2 in S. Newport; Incl1-IncFII(S)-IncFIB (S)-ColpVC in S. Typhimurium and Col440I in serovar 42:r:-. Nine sequence types, namely ST11, ST198, ST466, ST16, ST166, ST46, ST19, ST1208 and ST3807 were associated with S. Enteritidis, S. Kentucky, S. Zanzibar, 135 S. Virchow, S. Newport, S. Newport, S. Typhimurium, S. serovar 42:r:- and S. 136 Barranquilla, respectively.

L. 150: The percent prevalence of Salmonella (13.5%) in this study highlights the potential risk to humans to Ugandan households. This percentage is higher than that reported by Afema et al. (9) of 6.6% Salmonella in samples from live birds markets within Kampala, Uganda. [How meaningful is this comparison? Farms vs. live birds?] [Explain why the percentage might be higher.]

~~There was a seasonal effect in the recovery of Salmonella.~~ Uganda typically has a rainy season that occurs between March to May and October to December (10). Recovery of Salmonella was higher during the rainy season, possibly because the higher humidity and moisture allowed better dispersal or survival of Salmonella as observed for other bacterial species in poultry (11). Comparable to the situation in the US (12), Salmonella serovars Enteritidis and Kentucky were most often recovered. Serovar Kentucky has previously been reported in Uganda in humans, poultry, and the environment (9).

~~Among chicken isolates, Salmonella presented with MDR phenotypes to the 168 antimicrobials tested.~~ [New paragraph] Approximately 38% of the isolates were resistant to two or more classes of antimicrobials? [antibiotics?], including two isolates resistant to seven antimicrobials. The Salmonella serovar Kentucky isolates were resistant to over five (ciprofloxacin, nalidixic acid, streptomycin, sulfisoxazole, and tetracycline) or seven (chloramphenicol, ampicillin, ciprofloxacin, nalidixic acid, streptomycin, sulfisoxazole, tetracycline, and trimethoprim-sulfamethoxazole) antibiotics. All Salmonella serovar Kentucky isolates were resistant to ciprofloxacin. Since the early 2000s, ciprofloxacin resistant Salmonella serovar Kentucky have been on the rise, especially in travelers to northern and eastern Africa (13). Rickert-Hartman et al. (..??) found that 9% of the Salmonella serovar Kentucky isolated from travelers were ciprofloxacin resistant. Poultry was thought to be a reservoir for these resistant strains (13, 14). Ciprofloxacin-resistant Kentucky have caused seven persons to become ill and one death in the US after the carriers had travelled to India (13). In this regard, the emergence of S. Kentucky ST198 poses a major threat to public health worldwide since it is highly drug-resistant (15) and has been reported in different sources including retail chicken carcasses (16).

The presence of mutation can be useful for tracking the pandemic ciprofloxacin-resistant S. Kentucky strain ST198 from geographically distinct regions (15). Using WGS TEM-1B (beta lactamase?) was identified in five isolates that PCR methods did not identify. In previous studies (17), discrepancies were also seen between phenotypic resistance and genotypic analysis using WGS. It was reported that an MIC might not reach the breakpoint even though resistance genes were present (17). Five of the 28 plasmids that were screened through PCR were observed in multiple isolates: IncFIIS (17/51; 33.3%), IncI1α (12/51; 23.5%), IncP (8/51; 15.7%), 193 IncX1 (8/51; 15.7%), and IncX2 (1/51; 2.0%). After analyzing the WGS sequences for plasmids, 12 isolates were found to harbor IncI1α, with seven of the 12 having an additional plasmid (ColpVC) that was not detected by PCR and two with IncFIIS plasmid. Seventeen isolates carried the IncFIIS plasmid. These same 17 isolates also presented IncFIB (S) plasmids, and ColpVC and Col4401 were identified in seven and two isolates, respectively. IncX2 and IncP were not identified in the WGS analysis and by PCR. PCR did not detect plasmids in ten isolates, but WGS detected ColpVC 201 in nine isolates and Col4401 in one isolate. IncFIIS was the most common plasmid identified at 33.3% (17/51). Studies have shown that isolates containing blaCTX-M-1 harbor IncFIIS along with other incompatibility plasmids (18). Inc1 plasmids are known to be distributed throughout many serotypes of Salmonella and predominate in both E. coli and Salmonella 206 (19-21). In this study, Inc1α was observed among Salmonella serovars Zanzibar, Kentucky, and Typhimurium. All isolates from Salmonella serovar Kentucky and Typhimurium came from the same farm [what does this observation suggest?]. IncP and IncX1 were the next most common plasmids detected by PCR. Both were present in the Salmonella serovar Virchow isolates. It has been reported that IncP plasmids can spread via conjugative transfer and that they code for a broad range antimicrobial resistances. IncP is highly likely to be found in manure, wastewater, and soil (22). IncX1 is commonly found as a narrow host-range plasmid in Enterobacteriaceae, also spreading to other bacteria via conjugative transfer (23).

L. 216: In summary, we present in this study the clonal??[how do you know this?] distribution of eight Salmonella enterica serovars displaying resistance to clinically important antibiotics. Of these, the presence of international lineages such as ciprofloxacin-resistant S. Kentucky sequence type 198 in chicken farms raises a public concern given that fluoroquinolones are the first treatment choice. Our findings suggest that endemic dissemination of resistant serovars …[incomplete sentence], adding valuable information to the epidemiological surveillance in Uganda. Therefore, these results may encourage addition genomic surveillance studies in this region to aid in the development of mitigation strategies to limit the global distribution of these multidrug resistant Salmonella enterica.
