# Supplementary material for: International lineages of Salmonella enterica serovars isolated from chicken farms, Wakiso District, Uganda
Source: PLoS One. 2020 Jan 28;15(1):e0220484. doi: 10.1371/journal.pone.0220484 (PMC6986767; doi:10.1371/journal.pone.0220484)
Supplement: Attachment — Submitted filename: Response to editors 2.docx [file pone.0220484.s005.docx]

**Response to editors, Manuscript PONE-D-19-19750.R2**

6. Review Comments to the Author

Please use the space provided to explain your answers to the questions above. You may also include additional comments for the author, including concerns about dual publication, research ethics, or publication ethics. (Please upload your review as an attachment if it exceeds 20,000 characters)
**Response:** **We sincerely appreciate the editorial board and reviewers for their careful review and constructive suggestions. We believe that the manuscript was substantially improved after making the suggested edits. We have responded to specific queries below.**

Reviewer #3: (No Response)

Reviewer #5: The manuscript has been improved considerably.

**Response:** **We sincerely thank the reviewer for their favorable comments and interest in our work.**

The one comment I have now that Table 1 is available concerns n = 51. Is it possible that you included the same strains multiple times? The strains could have been isolated from different sites at a farm or even at different times. Would inclusion of the same strains not skew the percentages of resistance to particular antibiotics?

**Response:** **There were isolates collected on the same farms but at different sample sites (feed, water, litter, entryway, etc.) and different months. Most farms had new flocks in the area or in the process of getting new flocks between visits. The object of the entire study (*E. coli* and *Salmonella* for Cattle and Chicken) was to see if the prevalence and AMR results differ between the seasons and to identify what would be found. Every strain that was collected was presented in Table one to see all phenotypic and genotypic differences in both seasons. To avoid the percentages skew, we did not include the same strains multiple times, as mentioned above.**

Did the whole genome sequencing effort clearly demonstrate that all 51 isolates were different?

**Response: Although traditional tools have been considered the gold standards to study *Salmonella*, the whole-genome sequencing has been applied as an alternative to that after its establishment. In this regard, WGS recognizes antimicrobial resistance profile, MLST and evolutionary groupings that could precisely identify the differences between *Salmonella* strains. In this backdrop, we observed that the main drivers for cluster analysis were serotype, sequence types, and resistance profile since all isolates were clustered together by these characteristics and not by the period of isolation, source, or geographic location. We do have PFGE results not included in this manuscript for these isolates. They were not included due to the inclusion of other strains, not in this study. However, these PFGE results are in line with the WGS sequence data.**

Reviewer #6: Authors have revised paper in response to previous comments, but still need to address concerns. The over point of the study is to highlight the need for integrated surveillance for Salmonella using a One Health approach. I certainly concur with that, but think there is considerable restructuring of the paper needed to draw out that point.
In this relatively small data set, all of the MDR strains were S. Kentucky ST 198, which likely represent the expansion of a globally emerging strain. The authors note that these were from one farm that did not use fluoroquinolones. The spread of emerging strains across borders is a critical reason for integrated surveillance.
Specific comments:

Line 34 delete “all”

**Response:** **Addressed**

Line 61 does “chicken” imply live animals or poultry meat? Please clarify.

**Response: Live birds. Addressed**

Line 106 delete “all”

**Response:** **Addressed**

Line 139-140 It is important to expand on this analysis. 6/20 farms were positive in the dry season and 9/18 were positive during the wet season. The rate of Salmonella positivity appears to be about 9% for dry season, and 19% for rainy season. These are the data that are more interesting than the p-value that should be presented.

**Response: Remove the logistic regression statement.** **We agree that it is essential to expand this study. Indeed, these results may serve as a baseline study for future surveys.**

Line 142-145 It should be noted that all SE were pansusceptible, and all MDR isolates were S. Kentucky. The resistance data follow clonal strains of *Salmonella*.

**Response: Addressed**

Line 145 Table 2 does not include resistance data.

**Response:** **Addressed**

Line 170 Note ass are S. Kentucky

**Response:** **Addressed**

Line 209-213. See comments for lines 139-140. There is no point to mentioning the logistic regression if none of the output of the regression is presented.

**Response:** **Addressed**

Lines 182-208 There is a lot of detail presented here, but the context is not clear, and the reader easily gets lost. What is the bottom line? This is an opportunity to show how WGS can aid surveillance, but that does not clearly emerge from the presentation.

**Response: Statement was included “Although traditional tools have been considered the gold standard to study *Salmonella*, WGS has been applied as an alternative in providing more detailed and accurate data. In this regard, WGS recognizes antimicrobial resistance profile, MLST, and evolutionary groupings that could precisely identify the differences between *Salmonella* strains. We observed that the main drivers for characterization analysis were serotype, sequence types, and resistance profile. These isolates were clustered together by these characteristics and not by a period of isolation, source, or geographic location. To endorse these results, we have done pulsed-field gel electrophoreses (results not included), which are in agreement with the WGS results. Our study shows how WGS inspection constitutes a useful means to characterize *Salmonella* isolates.”**

Line 237-239 Does this mean that surveillance data for human illnesses were compared to results of this study? If so, that should be more thoroughly described and discussed. It would be useful to do so to highlight the One Health approach.

**Response:** **There were no human results compared in parallel to this study. The study mentioned in the manuscript was a project in Uganda looking at a few human cases, not for surveillance. The idea for our study was to get human data in parallel to compare, but resources were limited to do so. The statement in this line was implying that we did not see the same type of serovars as they saw in the Afema article. Clarification was added**

Line 258 and beyond… The presence of MDR Kentucky likely represents movement of clonal strains. This is the good justification of One Health integrated surveillance.

**Response:** **Addressed**
